# Native American Race, Use of the Indian Health Service, and Breast and Lung Cancer Survival in Florida, 1996–2007

**DOI:** 10.5888/pcd11.130162

**Published:** 2014-03-06

**Authors:** David J. Lee, Stacey L. Tannenbaum, Tulay Koru-Sengul, Feng Miao, Wei Zhao, Margaret M. Byrne

**Affiliations:** Author Affiliations: Stacey L. Tannenbaum, Feng Miao, Wei Zhao, Sylvester Comprehensive Cancer Center University of Miami Miller School of Medicine, Miami, Florida; Tulay Koru-Sengul, Margaret M. Byrne, University of Miami Miller School of Medicine Department of Public Health Sciences and Sylvester Comprehensive Cancer Center, Miami, Florida.

## Abstract

We evaluated associations of race, primary payer at diagnosis, and survival among patients diagnosed in Florida with lung cancer (n = 148,140) and breast cancer (n = 111,795), from 1996 through 2007. In multivariate models adjusted for comorbidities, tumor characteristics, and treatment factors, breast cancer survival was worse for Native American women than for white women (hazard ratio [HR], 1.52; 95% confidence interval [CI], 1.05–2.20) and for women using the Indian Health Service than for women using private insurance (HR, 1.71; 95% CI, 1.33–2.19). No survival association was found for Native American compared with white lung cancer patients or those using the Indian Health Service versus private insurance in fully adjusted models. Additional resources are needed to improve surveillance strategies and to reduce cancer burden in these populations.

## Objective

The Indian Health Service (IHS) relationship was initially established in 1787 but formally recognized in 1955 as the principal federal health care provider and health advocate for Native Americans (1,2); the goal of IHS health services is to optimize the health status of Native Americans. However, long-standing health disparities between Native Americans and the general US population exist (1). For example, the life expectancy of Native Americans is shorter than that of whites (71.5 y vs 75.6 y) (1). Conversely, mortality rates per 100,000 are lower in Native Americans than in the general population for cancers of the lung (43.0 vs 66.7) and breast (9.8 vs 17.7) (3). However, limited research has documented associations among Native American race, use of IHS, and survival time after cancer diagnosis (4). In this study, we used the Florida Cancer Data System (FCDS), a Florida population-based cancer registry, to examine breast and lung cancer survival by Native American race and IHS use for the Seminole and the Miccosukee tribes, the 2 federally recognized tribes in Florida.

## Methods

FCDS data (1996–2007) were linked with data from the Florida Agency for Health Care Administration (AHCA). Incident lung cancer and female breast cancer were identified from the FCDS. FCDS collects information on diagnosis, stage, demographics, treatments, primary payer at diagnosis, and date of death (5). Patients were categorized as IHS users for primary payer at diagnosis if they reported using IHS services in FCDS. AHCA data contain medical records on all patients treated at hospitals and free-standing surgical and radiological treatment centers (6).

The primary outcome of our study, overall survival, was elapsed time from diagnosis to date of death or last patient encounter. Our main predictors of interest were race (white, Native American, black, Asian, Pacific Islander, Asian Indian/Pakistani, or other) and primary payer at diagnosis (private, IHS, Medicaid, Medicare, defense/military/veteran, insurance not otherwise specified, or uninsured). However, we focused primarily on Native Americans versus whites and IHS versus private insurance. We excluded non-Florida residents aged 18 years or younger, patients with missing values for survival time, and patients with carcinoma in situ.

We used Cox proportional hazards regression models to obtain hazard ratios (HRs) and 95% confidence intervals (CIs) by cancer type from 4 models (univariate, multivariate not adjusted for race, not adjusted for IHS, and fully adjusted). This project was approved by the University of Miami’s institutional review board.

## Results

Of 238, 427 patients who met our study criteria, 41 lung cancer patients and 38 breast cancer patients self-reported as Native American; however, 176 lung cancer patients and 177 breast cancer patients reported using IHS providers (Table 1). Native Americans with breast and lung cancer were younger than their white counterparts; we found more than a 6-year difference in mean age among women with breast cancer (Native Americans, 57.5 y vs whites, 64.1 y). Patients using IHS were younger than those using private insurance; we found larger differences among lung cancer patients (58.7 y, IHS vs 64.0 y, private insurance) (Table 2).

**Table 1:** Sociodemographic Characteristics of White and Native American Breast and Lung Cancer Patients (N = 238,427), Florida, 1996–2007

Characteristic	Breast Cancer		Lung Cancer	
	Native American (n = 38)	White (n = 101,517)	Native American (n = 41)	White (n = 136,831)
**Mean age, y (SD)**	57.5 (13.0)	64.1 (13.9)	67.1 (11.8)	69.9 (10.9)	
**Sex**					
Male	NA	NA	29	74,915	
Female	38	101,517	12	61,916	
**Race**					
White	—	101,517	—	136,831	
Native American	38	—	41	—	
**Primary payer at diagnosis**					
Indian Health Service	3	174	0	176	
Private insurance	14	35,664	8	28,547	
Medicaid	2	2,560	2	4,585	
Medicare	15	46,928	23	86,654	
Defense/military/veteran	0	1,236	0	2,300	
Insurance not otherwise specified	3	11,913	5	9,980	
Uninsured	1	3,042	3	4,589	
**Marital status**					
Never married	3	9,539	4	13,060	
Divorced/separated/widowed	14	32,146	7	41,622	
Married	19	57,251	24	78,870	
Unknown	2	2,581	6	3,279	
**Socioeconomic status^a^**					
Low	10	8,058	7	12,973	
Middle low	16	29,433	14	44,485	
Middle high	7	40,665	15	54,161	
High	5	23,361	5	25,212	
**Tobacco use**					
Never smoked	19	49,175	4	11,798	
History of smoking	5	20,052	17	59,987	
Current smoker	8	13,339	15	49,185	
Unknown	6	18,951	5	15,861	
**Urban/rural**					
Urban	32	96,092	36	127,301	
Rural	6	5,425	5	9,530	
**Hospital volume**					
Low	19	60,571	30	93,726	
High	19	40,946	11	43,105	
**Type of health care facility**					
Nonteaching	28	91,911	39	126,862	
Teaching	10	9,606	2	9,969	

a Neighborhood area poverty levels derived from the US Census and characterized into 4 groups by percentage of a neighborhood living in poverty.

**Table 2:** Sociodemographic Characteristics of Breast and Lung Cancer Patients by Primary Payer at Diagnosis, Florida, 1996–2007

Characteristic	Breast Cancer		Lung Cancer	
	Indian Health Service (n = 177)	Private ( n = 35,678)	Indian Health Service (n = 176)	Private (n = 28,555)
**Mean age, y (SD)**	52.2 (10.2)	55.9 (12.0)	58.7 (11.2)	64.0 (11.2)
**Sex**				
Male	—	—	89	15,284
Female	177	35,678	87	13,271
**Race**				
White	174	35,664	176	28,547
Native American	3	14	0	8
**Marital status**				
Never married	46	3,993	44	2,995
Divorced/separated/widowed	59	7,851	73	6,820
Married	66	23,024	56	18,143
Unknown	6	810	3	597
**Socioeconomic status^a^**				
Low	33	2,355	31	2,414
Middle low	69	9,658	62	9,207
Middle high	51	14,248	54	11,489
High	24	9,417	29	5,445
**Tobacco use**				
Never smoked	65	17,148	7	2,339
History of smoking	26	6,452	50	11,080
Current smoker	47	5,357	106	11,778
Unknown	39	6,721	13	3,358
**Urban/rural**				
Urban	175	34,624	171	27,697
Rural	2	1,054	5	858
**Hospital volume**				
Low	54	19,649	96	19,037
High	123	16,029	80	9,518
**Type of health care facility**				
Nonteaching	147	31,999	167	26,346
Teaching	30	3,679	9	2,209

a Neighborhood area poverty levels derived from the US Census and characterized into 4 groups by percentage of a neighborhood living in poverty.

For female breast cancer patients, Native American race was not significant in the univariate model (HR, 1.38; 95% CI, 0.93–2.06) (Table 3). But in multivariate models, Native Americans (not adjusted for primary payer) had worse survival than whites (HR, 1.48; 95% CI, 1.03–2.12); in the fully adjusted model, Native Americans maintained worse survival than whites (HR, 1.52; 95% CI, 1.05–2.20). Breast cancer patients using IHS had worse survival than those using private insurance in the univariate model (HR, 1.73; 95% CI, 1.43–2.11) and in the multivariate model without adjustment for race (HR, 1.76; 95% CI, 1.36–2.27); this survival disadvantage was maintained in the fully adjusted model (HR, 1.71; 95% CI, 1.33–2.19).

**Table 3:** Association of Cancer Survival With Native American Race and Use of Indian Health Service as Primary Payer^a^, Florida, 1996–2007

Model	Factor	Breast Cancer, Hazard Ratio (95% CI)	Lung Cancer, Hazard Ratio (95% CI)
**Univariate**	Native American vs white	1.38 (0.93–2.06)	1.08 (0.76–1.53)
	IHS vs private	1.73 (1.43–2.11)	1.25 (1.08–1.44)
**Multivariate**			
Fully adjusted except primary payer designation	Native American vs white	1.48 (1.03–2.12)	0.98 (0.71–1.37)
Fully adjusted except race	IHS vs private	1.76 (1.36–2.27)	1.21 (0.99–1.49)
Fully adjusted	Native American vs white	1.52 (1.05–2.20)	0.98 (0.71–1.36)
	IHS vs private	1.71 (1.33–2.19)	1.21 (0.99–1.49)

Abbreviations: CI, confidence interval; IHS, Indian Health Service.

a Other race designations (black, Asian, Pacific Islander, Asian Indian or Pakistani, and other) and other types of primary payers at diagnosis (Medicaid, Medicare, defense/ military/veteran, insurance not otherwise specified, and uninsured) were included in the model but not shown here. Fully adjusted models included age; other races; other types of primary payers at diagnosis; ethnicity (Hispanic or non-Hispanic); sex (for lung cancer); neighborhood area poverty levels derived from the US Census and characterized into 4 groups by percentage of neighborhood living in poverty, marital status, smoking status, comorbidities; and cancer-related indicators (tumor grade and stage, lymph node status, type of treatments, histology).

In the univariate model, lung cancer patients using IHS had worse survival than those using private insurance (HR, 1.25; 95% CI, 1.08–1.44), but Native Americans patients did not have worse survival than whites (HR, 1.08; 95% CI, 0.76–1.53). We found no significant survival differences between Native Americans and whites or IHS use and private insurance in any adjusted models.

## Discussion

Our study found that Native American race and use of IHS were independent predictors of survival among women diagnosed with breast cancer but not for people diagnosed with lung cancer. We also documented little association between Native American race and use of IHS; for example, only 3 Native Americans reported receiving health care from IHS. This apparent discrepancy possibly arises from people self-reporting race as non-Native American when they are of mixed Native American and other race. Incorrect or incomplete classification of Native American race has been documented in other health surveillance systems and needs to be addressed to characterize the diverse Native American population more accurately in cancer registries (7). Conversely, some research has found high levels of agreement between self-reported Native American race and administrative data (8). Researchers cannot assume that race is accurately reported. Given documented social and economic disadvantages as well as diverse cultural practices among members of the Native American community, our findings, like those of others, raise the question of whether current cancer care is adequate to meet the needs of this community (3). For example, some cancer care costs such as specialized imaging studies may not be provided by IHS, in part because of chronic program underfunding by appropriations from Congress (9).

Although our study controls for numerous factors, it cannot identify small differences in quality of cancer care. Racial discrimination and its role in receipt of high-quality cancer care may be a factor in reduced survival, given evidence of its adverse influence on cancer screening behaviors in Native American communities (10). Other factors that may affect survival for Native Americans with lung and female breast cancer include mistrust of the medical community, patient–provider miscommunication, and access to care.

A limitation of our study is that it may not reflect the mortality among Native American groups residing outside of Florida. For example, breast cancer mortality rates range from 7.4 to 11.6 per 100,000 across IHS regions (11). Although promising work using patient navigators to improve cancer prevention, early detection, and cancer treatment outcomes is underway (12), financial support for such activities is limited relative to unmet needs of this population. Our study supports calls for additional resources to improve surveillance strategies and reduce cancer burden in this population (12,13).
